# Glutathione in Our Diet and Its Role in the Body: From Disease Prevention to Anti-Aging

**DOI:** 10.3390/nu18101640

**Published:** 2026-05-21

**Authors:** Vijolė Bradauskienė, Elena Moščenkova, Gražina Šniepienė, Reda Kubiliūtė, Lina Vaičiulytė

**Affiliations:** 1 Department of Computer Science and Biotechnology, Klaipėdos valstybinė kolegija/Higher Education Institution, Bijunu Street 10, LT-91223 Klaipeda, Lithuania; e.moscenkova@kvk.lt (E.M.); g.sniepiene@kvk.lt (G.Š.); 2 Faculty of Health Sciences, Klaipėda University, Herkaus Manto Street 84, LT-92294 Klaipeda, Lithuania; reda.dzingeleviciene@ku.lt; 3 Food Institute, Kaunas University of Technology, Radvilenu Road 19C-413, LT-50254 Kaunas, Lithuania; lina.vaiciulyte@ktu.lt

**Keywords:** glutathione, antioxidant, detoxification, anti-aging

## Abstract

**Background/Objectives**: Glutathione (GSH) is a fundamental tripeptide essential for maintaining cellular redox homeostasis, detoxification, and immune regulation. While GSH is synthesized endogenously, its levels typically decline with age, potentially increasing susceptibility to oxidative stress-related conditions. This review aims to discuss the benefits of GSH for the body and clarify the distinctions between dietary intake, endogenous synthesis, and supplementation as strategies for maintaining optimal GSH levels. **Results**: All studies show that GSH is a powerful antioxidant that plays a crucial role in maintaining various physiological processes in the body. It offers several benefits, primarily through its antioxidant properties and involvement in detoxification and immune regulation. This effect has potential implications for various health conditions associated with oxidative stress and inflammation, including neurodegenerative diseases, cardiovascular diseases, and metabolic disorders. Whether through diet or supplementation, ensuring adequate GSH levels can have profound benefits on longevity, immunity, and overall well-being. There are many foods known to contain GSH, and there are also many GSH supplements available on the market, but precursor-based supplements and compounds that activate GSH synthesis pathways show stronger and more consistent increases in human GSH. A diet rich in protein (for amino acids) and phytochemical-dense plants can support this, while targeted precursors (e.g., glycine, γ-glutamylcysteine) and Nrf2-activating foods or agents provide the most robust increases shown so far. Such supplementation can be beneficial, and it is most effective when combined with a diet rich in sulfur-containing foods and other nutrients that support GSH synthesis.

## 1. Introduction

Glutathione (GSH) is an important substance that is naturally produced in our bodies. However, due to lifestyle and other factors, GSH levels decrease with age, weakening the body’s protection against oxidative stress, toxins, and viruses. Many manufacturers currently market GSH supplements, often promoted for anti-aging properties. However, limited public understanding exists regarding supplement effectiveness, bioavailability differences between approaches, and whether dietary strategies alone are sufficient. This review evaluates current scientific evidence on GSH and its effects on human health and also addresses three critical questions: (1) Does the body have a GSH deficiency? (2) Can a balanced diet provide adequate GSH levels? (3) For which populations and conditions are supplementation indicated, and which approaches (direct GSH versus precursor-based) demonstrate greater efficacy?

A key focus is distinguishing three approaches to maintaining adequate GSH levels: (1) dietary intake of GSH through foods, (2) endogenous synthesis stimulated by dietary precursors and bioactive compounds, and (3) supplementation with GSH or GSH precursor compounds. These approaches differ significantly in efficacy, bioavailability, and clinical appropriateness.

The structure of GSH is well known and it determines its specific properties. Its chemical formula is C_10_H_17_N_3_O_6_S. GSH is a tripeptide composed of three amino acids: glutamic acid, cysteine, and glycine (γ-Glu-Cys-Gly) [1]. The structure of GSH is characterized by a thioether bond between the cysteine and glutamic acid residues, which gives it its special properties. The cysteine thiol (–SH) provides reducing power and radical scavenging ability; its oxidation to disulfide (GSSG) underlies the GSH/GSSG redox couple central to cellular redox homeostasis) [1,2,3]. The thiol’s nucleophilicity enables conjugation to electrophilic toxins and drug metabolites, either spontaneously or via glutathione S transferases [2,4]. Carboxyl and amino groups across glutamate and glycine provide metal binding and electrophile binding sites, supporting metal detoxification, phytochelatin synthesis in plants, and drug metabolite trapping [4,5]. GSH’s activity is not due to the thiol alone: the γ-linked tripeptide scaffold and multiple coordinating groups make it stable, abundant, enzyme recognizable, and highly reactive in the right way, enabling its central roles in redox control, detoxification, and signaling across organisms [5].

**Figure 1:**
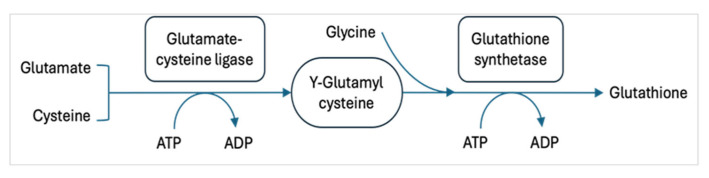
Synthesis of GSH. GSH synthesis is a two-step process where Glu, Cys, and Gly are catalyzed in the presence of enzymes glutamate-cysteine ligase and glutathione synthetase.

GSH is synthesized in the body through a two-step enzymatic process that occurs in the cytosol of cells. This synthesis is essential for maintaining cellular redox balance and detoxification. The initial step in GSH synthesis is catalyzed by the enzyme glutamate-cysteine ligase (Figure 1). This rate-limiting step involves the combination of glutamate and cysteine to form γ-glutamylcysteine [6]. This process requires energy in the form of ATP [7,8]. The second step, formation of GSH, is catalyzed by glutathione synthetase, which combines γ-glutamylcysteine with glycine to produce GSH. This step also requires ATP [7,8].

The synthesis of GSH is tightly regulated, with the availability of cysteine often being a limiting factor; hence, a shortage of cysteine can lead to reduced GSH production [8]. The second factor is enzyme regulation: the expression and activity of the enzymes involved in GSH synthesis can be influenced by various physiological and pathological conditions, which can impair GSH homeostasis [7,9]. Hence, GSH synthesis is a critical biochemical process involving two ATP-dependent enzymatic steps. Understanding these mechanisms is important for addressing conditions that affect GSH levels, such as liver diseases and oxidative stress. The liver plays a central role in GSH synthesis and distribution to other organs [7,10]. GSH is synthesized in the cytosol but needs to be transported to various organelles and tissues and the liver is particularly important in maintaining systemic GSH levels through interorgan transport [10,11].

## 2. Materials and Methods

### 2.1. Literature Search Strategy and Selection Criteria

A systematic literature search was conducted in PubMed, Taylor & Francis Online, and Google Scholar databases covering the period 2015 to March 2026. The search employed the keywords “glutathione” combined with “benefits,” “antioxidant,” “detoxification,” “disease prevention,” “anti-aging,” and “food sources.” The search was restricted to publications in English.

### 2.2. Inclusion and Exclusion Criteria

Inclusion criteria were: (1) peer-reviewed original research articles, systematic reviews, meta-analyses, or clinical trials; (2) studies addressing GSH biochemistry, functions, dietary sources, bioavailability, or supplementation; (3) English-language publications; (4) data on human, animal, or in vitro studies clearly identified as such.

Exclusion criteria were: (1) non-peer-reviewed articles or opinion pieces without data; (2) studies lacking clear methodology or relevant GSH data; (3) duplicate publications; (4) abstracts without full-text availability.

### 2.3. Article Selection and Analysis

The initial search identified 388 articles. After reviewing titles, abstracts, and full texts against inclusion criteria, 109 articles were retained for comprehensive analysis. Additional relevant publications on GSH food sources and dietary strategies were identified through cross-referencing. Data were extracted regarding GSH biochemistry, physiological functions, dietary sources, precursor strategies, supplementation efficacy, bioavailability, and safety. Analysis emphasizes distinguishing evidence quality from in vitro studies, animal models, and human clinical trials. Results are presented using descriptive analysis organized by GSH functions and approach.

## 3. Results and Discussion

### 3.1. Benefits of Glutathione for the Body and Role in Disease Prevention

**Figure 2:**
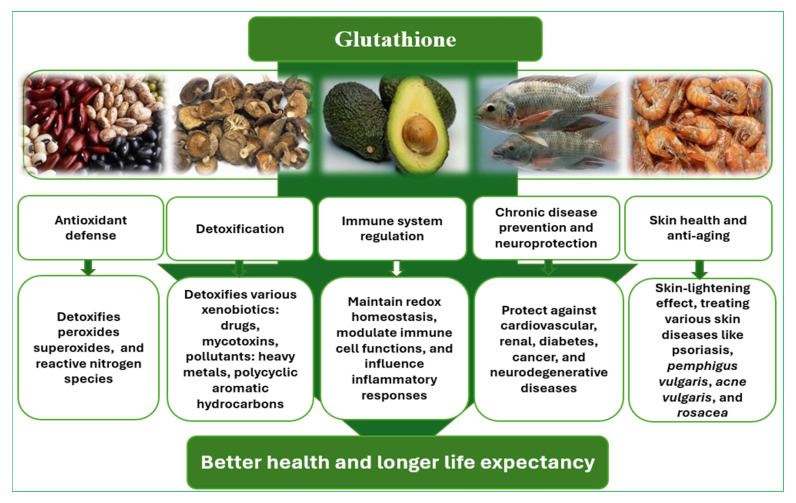
Benefits of glutathione for the body.

It is well known that GSH is one of the most powerful antioxidants and has a wide range of health benefits. GSH is involved in detoxifying reactive oxygen species (ROS) and xenobiotics, maintaining redox homeostasis, regulating cell growth and apoptosis [7,10,12]. It is also crucial in the brain for protecting against oxidative stress and is implicated in neurodegenerative diseases [13,14]. Based on the latest scientific evidence the potential benefits of GSH to the body can be categorized into several areas which are represented graphically in Figure 2.

#### 3.1.1. Antioxidant Defense

GSH is involved in detoxifying reactive oxygen species (ROS), which include peroxides and superoxides, thereby protecting biological membranes from lipid peroxidation and maintaining cellular homeostasis [10,15]. GSH is also essential for neutralizing reactive nitrogen species (RNS), thereby reducing oxidative stress and protecting cells from damage [3,12,16]. In specific therapeutic contexts, such as tumor treatments, GSH depletion is used to enhance the accumulation of alkyl radicals, which are otherwise neutralized by GSH [17]. The sulfhydryl (thiol) groups in GSH are critical for their role in neutralizing free radicals and reducing peroxides, contributing to detoxification and cellular signaling processes. GSH works in conjunction with enzymes like glutathione peroxidase and glutathione S-transferase to enhance its antioxidant capacity and facilitate the detoxification process [18].

#### 3.1.2. Detoxification

GSH is involved in detoxifying xenobiotics and heavy metals, protecting the body from these harmful substances [19]. GSH acts as a nucleophilic scavenger, neutralizing electrophilic centers in various xenobiotics, thus preventing cellular damage. GSH detoxifies various xenobiotics and their metabolites, including drugs, pollutants, and carcinogens, by neutralizing electrophilic compounds that can form harmful adducts with cellular macromolecules [10,20]. GSH also helps neutralize oxidative stress induced by mycotoxins such as T-2 toxin, deoxynivalenol, and fumonisin B1, which are commonly found in feed commodities [21]. Another redox toxin, Pyocyanin, produced by *Pseudomonas aeruginosa* could be detoxified by GSH, which forms a non-toxic conjugate with pyocyanin, reducing its cytotoxicity [22]. GSH is involved in the detoxification of polycyclic aromatic hydrocarbons like phenanthrene and benzo[b]fluoranthene. These compounds increase ROS production, and GSH levels are upregulated to protect against oxidative damage. The GSH system helps modulate the toxicity of these substances through mechanisms like protein-S-glutathionylation [23]. Although GSH weakly binds to heavy metals, like lead (Pb), it plays a role in detoxifying these metals too. The detoxification capabilities of GSH can be enhanced by modifications such as thiolation and carboxylation, which improve its ability to bind and neutralize metals [24].

#### 3.1.3. Immune System Regulation

GSH plays a role in regulating the immune system, which can help in maintaining overall health and potentially in preventing age-related diseases [12,25]. GSH regulates the immune system by maintaining redox homeostasis, modulating immune cell functions, and influencing inflammatory responses [26]. GSH is essential for the function of regulatory T cells (Tregs), which maintain immune homeostasis and prevent autoimmunity. GSH restricts serine metabolism, preserving T cell functionality and suppressive capacity, which is crucial for preventing autoimmune diseases and regulating anti-tumor responses [27,28]. GSH influences the activation of some inflammasomes, a component involved in immune and inflammatory responses. GSH metabolism contributes to the induction of trained immunity, characterized by enhanced responses to secondary challenges. It modulates pro-inflammatory cytokine production in monocytes, indicating its role in immune memory and response [29].

As a major antioxidant, GSH prevents oxidative damage in immune cells, such as macrophages and T cells, by stabilizing redox activity and modulating cytokine profiles. This is particularly important in infections like tuberculosis, where GSH enhances immune responses [30]. GSH affects immune responses associated with fever by modulating oxidative stress. It can reduce or inhibit fever, indicating its role in managing immune reactions during infections [31]. Hence GSH is integral to immune system regulation through its roles in maintaining redox balance, modulating immune cell functions, and influencing inflammatory pathways. Its involvement in Treg functionality, inflammasome activation, and trained immunity underscores its importance in both innate and adaptive immune responses. Therefore, these functions make GSH a potential target for therapeutic strategies in managing immune-related diseases and altered GSH levels are linked to various pathologies, including autoimmune disorders, highlighting its regulatory role in inflammation [32].

#### 3.1.4. Mitochondrial Protection

GSH supports mitochondrial metabolism, which is crucial for energy production and reducing oxidative damage in cells [3,19]. GSH is vital for mitochondrial protection by maintaining redox balance, regulating mitochondrial dynamics, and preventing oxidative stress. Its import and homeostasis are tightly regulated to ensure effective defense against cellular damage. These protective roles are crucial for preventing mitochondrial dysfunction and associated diseases. GSH plays a crucial role in maintaining mitochondrial function by acting as a key antioxidant that helps regulate oxidative stress and redox balance within the mitochondria, thereby preventing oxidative damage and maintaining cellular health. It metabolizes hydrogen peroxide and other ROS, reducing oxidative stress and preventing mitochondrial dysfunction [33,34].

Stimulation of GSH synthesis has been shown to improve mitochondrial efficiency by increasing mitochondrial membrane potential and reducing oxygen consumption. This result in decreased ROS production and improved respiratory chain efficacy was determined in aging animal and human models [35,36]. GSH maintains the redox balance within mitochondria, which is crucial for preventing cell dysfunction and death. In this way, an imbalance in mitochondrial ROS and GSH levels can lead to cell apoptosis and necroptosis, contributing to various diseases [33,34].

GSH is involved in the biosynthesis and export of iron–sulfur clusters, which are vital for mitochondrial function and cellular iron metabolism [37]. GSH reduces oxidative stress markers and prevents the opening of the mitochondrial permeability transition pore, which is crucial for protecting against ischemia/reperfusion injury and other oxidative stress-related damages [36,38,39]. Mitochondria have feedback mechanisms to regulate GSH levels, ensuring adequate protection against oxidative stress. This involves the regulation of transport proteins and their interaction with mitochondrial proteases [5,40]. It plays a role in energy metabolism, ROS production, and apoptosis [41].

#### 3.1.5. Chronic Disease Prevention

Several studies involving animals and humans have shown that low levels of GSH are associated with chronic conditions such as cardiovascular, renal, and neurodegenerative diseases. Supplementation may improve outcomes in these conditions [19,42]. Research indicates that GSH may play a role in improving insulin sensitivity, which is crucial for preventing type 2 diabetes. Low levels of GSH have been associated with insulin resistance, highlighting its importance in metabolic health. Elevated oxidative stress is linked to insulin resistance; thus, maintaining adequate GSH levels can help mitigate oxidative damage to insulin signaling pathways, enhancing insulin sensitivity. Some studies have shown that increasing GSH levels may improve insulin sensitivity and prevent diet-induced obesity [43]. By reducing oxidative stress, GSH aids in preserving the integrity of the insulin signaling cascade. This includes the activation of key proteins involved in glucose uptake, such as the insulin receptor substrate and protein kinase B, which are essential for effective insulin action [44,45]. Additionally, compounds that boost GSH levels or mimic its action have been explored as potential therapeutic strategies for improving metabolic health.

GSH serves as a critical antioxidant that protects against cancer by detoxifying harmful substances and maintaining redox balance. This protection is vital for preventing cellular damage that can lead to cancer development [46,47]. GSH conjugates with xenobiotics, including carcinogens, to form more water-soluble compounds that are easily excreted, reducing the likelihood of these substances causing cellular damage [48]. On the other hand, its elevated levels in cancer cells can promote tumor survival and drug resistance, presenting both challenges and opportunities for cancer therapy. Targeting GSH pathways may enhance the efficacy of existing treatments and overcome drug resistance. In this way, GSH plays both protective and negative roles in cancer, as elevated GSH levels in cancer cells help maintain redox homeostasis, supporting cell survival and proliferation under oxidative stress conditions [49,50]. High GSH levels in tumor cells are associated with increased resistance to chemotherapy, as GSH can neutralize the ROS generated by anticancer drugs, reducing their efficacy [50,51]. GSH is involved in key cellular processes such as cell differentiation, proliferation, and apoptosis, which can contribute to tumor progression when dysregulated [50]. That is precisely why cancer therapy uses a reverse strategy to reduce GSH in cancer cells, which increases the efficacy of ROS-based therapies and chemotherapy by making cancer cells more sensitive to oxidative stress [49,51,52].

Some studies also suggest a positive correlation between GSH levels and vitamin D production in the body. Adequate GSH may enhance the expression of genes involved in positive vitamin D metabolism, which is linked to the reduction in various chronic diseases, including cardiovascular disease and diabetes [53]. In general, it can be stated that GSH acts as a powerful protector against oxidative stress, aids in detoxification processes, supports immune function, and contributes to metabolic health, and these functions collectively contribute to its role in preventing various chronic diseases.

#### 3.1.6. Neuroprotection

The protective roles of GSH in the brain suggest potential therapeutic avenues for neurodegenerative diseases characterized by oxidative stress, such as Alzheimer’s disease and Parkinson’s disease. Increasing GSH levels or enhancing its activity could be beneficial strategies for neuroprotection. GSH is important for brain health, potentially offering protection against neurodegenerative diseases by enhancing mitochondrial function and reducing oxidative stress [3]. GSH is essential in detoxifying ROS and RNS, thereby preventing oxidative damage and inflammation in neuronal cells. This is particularly important in the brain, which is highly susceptible to oxidative stress due to its high oxygen consumption [13,54]. GSH is involved in regenerating other antioxidants, such as vitamins C and E, enhancing the brain’s overall antioxidant capacity. GSH helps in maintaining mitochondrial function, regulating metal homeostasis, and supporting processes like autophagy and apoptosis, which are vital for neuronal survival and function. By supporting mitochondrial function, GSH helps ensure that neurons have adequate energy to perform their functions, which is crucial for maintaining cognitive processes [14]. GSH interacts with glutamate, a neurotransmitter that, in excess, can lead to excitotoxicity and neuronal death. Increased levels of GSH during glutamate-mediated neuronal activity help protect neurons by reducing oxidative stress associated with excitatory overdrive [54]. Therefore, GSH protects the brain primarily through its antioxidant properties, regulation of excitatory neurotransmitter activity, and support for mitochondrial function. These mechanisms collectively contribute to neuronal survival and cognitive health.

#### 3.1.7. Skin Health and Effects on Aging Markers

GSH has been investigated for skin-lightening properties and potential therapeutic effects. Oral administration of GSH has been shown to improve skin health and reduce signs of aging [55]. It affects the skin primarily through its anti-melanogenic properties, which help in reducing pigmentation and lightening skin tone. GSH inhibits the enzyme tyrosinase, which is crucial in melanin production, thereby reducing pigmentation and promoting skin lightening [56,57]. GSH shifts melanin synthesis towards producing lighter pheomelanin instead of darker eumelanin, contributing to its skin-lightening effect in this way [56]. GSH has also been found useful in treating various skin diseases like psoriasis, *pemphigus vulgaris*, *acne vulgaris*, and *rosacea* [58]. As an antioxidant, GSH helps protect the skin from oxidative stress, which can contribute to hyperpigmentation and other skin issues [57,58,59]. Another study [60] showed that GSH yielded other cosmetic benefits as it may improve skin elasticity and reduce skin wrinkles.

Some studies suggest that GSH may support hair growth by promoting a healthy environment for hair follicles. Its role in reducing inflammation and supporting cellular health can be beneficial for maintaining strong and vibrant hair [61]. However, direct evidence linking GSH supplementation specifically to enhanced hair beauty is still limited.

GSH is frequently described in popular media and marketing as an “anti-aging” agent. While animal models demonstrate associations between GSH and longevity, human evidence for lifespan extension is absent. Most relevant human data to date suggest that GSH supports “healthy aging biology,” but there is a lack of evidence that it extends human lifespan. Most human data comes not from direct use of GSH tablets, but from GlyNAC (glycine + *N*-acetylcysteine), which increases intracellular GSH levels. In a 16-week RCT with older adults, GlyNAC corrected GSH deficiency, reduced oxidative stress, and improved mitochondrial function, inflammation, insulin resistance, endothelial function, and many “signs of aging”, as well as improved walking speed, strength, and 6 min walk distance [62,63,64,65], leading to the conclusion that GlyNAC reverses many age-related impairments, improving the health of aging individuals, though lifespan was not assessed [62,65].

Thus, some studies found that GSH improves markers of metabolic age. Recent scientific reviews refer to GSH as “an effective therapeutic agent for the treatment of age-related diseases” and highlight its proven links to lifespan in animals, but they emphasize that there is a lack of direct evidence that it extends life or slows aging in humans, and that long-term studies are needed [12,66,67].

Thus, the scientific literature provides ample evidence that GSH is crucial for human health, but it is also important to address the question of how to ensure adequate levels of it in the body. Three approaches exist for supporting GSH: dietary intake, endogenous synthesis stimulation, and supplementation.

### 3.2. The Role of Diet in Increasing GSH Levels

GSH levels in the body were previously thought to be independent of diet, but in 2015 it was found that certain foods can increase them [68]. Dietary GSH intake is linked to plasma GSH levels, but factors regulating plasma GSH concentration are complex and related to dietary GSH or precursor amino acids intake [68].

#### 3.2.1. The Best Dietary Sources of GSH

GSH is found in various foods. The best dietary sources of GSH include certain fresh fruits, vegetables, mushrooms, meats and seafoods (see Table 1).

Fresh fruits and vegetables generally contain moderate to high levels of GSH, ranging, on average, from 4.0 to 15.0 mg/100 g wet weight [72]. There is sufficient research to show that legumes accumulate high levels of GSH, especially when grown under stressful conditions. However, most studies evaluate GSH as a plant metabolite and examine it in various parts of the plant (µmol per gram of tissue) rather than as a food nutrient. From the available data, it can be calculated that legumes (mung beans, black beans, kidney beans, pinto beans, etc.) may contain GSH about 15.00–37.00 mg/100 g [70,71] and provide a moderate amount of GSH along with other essential nutrients. Mushrooms can be an excellent dietary source of GSH too. Certain species of mushrooms, such as *Agaricus bisporus*, known as Portobello mushroom or cultivated mushroom (champignons), are particularly high in GSH, with levels varying significantly among different types (11.00–241.00 mg/100 g of dried product) [69].

Some studies have found that some types of fish, shrimp, and other seafood contain high levels of GSH [76,77,78] while food groups of dairy products and cereals generally have low levels of GSH, making them less significant sources. Freshly prepared meats are also relatively high in GSH, with contents ranging from 5.0 to 20.0 mg/100 g wet weight. Processing and cooking often lead to a significant reduction in GSH content, so fresh and minimally processed foods are preferable [83].

It should be noted that although certain foods contain measurable quantities of GSH, the actual bioavailability of dietary GSH remains quite low [84]. The concentration of GSH in foods varies considerably depending on storage conditions, processing methods, and preparation techniques, with heat treatment and oxidative stress generally reducing GSH content.

#### 3.2.2. Foods to Increase Dietary Glutathione

GSH‘s levels can be influenced by other dietary sources. While direct GSH supplementation is not very effective due to its degradation in the gut, certain foods and nutrients can help increase its levels indirectly, boosting endogenous synthesis via precursors or pathway activation [84,85,86]. Various foods containing sulfur and amino acids like cysteine and glycine, spices, and herbs like *Allium hookeri*, and Brassica vegetables have been shown to stimulate GSH production and improve antioxidant status. Table 2 summarizes foods that stimulate GSH production in the body.

Most studies proved that amino acids cysteine, glycine and glutamate, which are direct precursors of GSH, are essential for its synthesis; therefore, foods with these amino acids can increase GSH synthesis in tissues [25,86]. Sulfur-rich foods like garlic and onions also support the synthesis of GSH in the body. Although legumes provide a moderate amount of GSH, they contain other biologically active compounds and elements that contribute to GSH synthesis in the body in various ways [97,99].

Some studies show that certain plant extracts and spices can activate GSH synthesis in the body. For example, curcumin supplementation may lead to increased plasma and tissue levels of GSH [101]. Curcumin influences GSH levels by acting as an antioxidant, promoting its synthesis, regulating cellular redox status, and reducing inflammation. These mechanisms collectively contribute to enhanced GSH availability in the body. Other phytochemicals found in foods can influence GSH levels too. For example, resveratrol [89] or green tea polyphenols [91] have been shown to improve GSH status and overall antioxidant capacity. Expression of γ-glutamyl cysteine ligase, which facilitates cystine uptake, is regulated by nuclear factor erythroid-2-related factor 2 (Nrf2). Nrf2 activating compounds (e.g., sulforaphane, dithiolethiones) upregulate GSH synthesis enzymes and increase GSH level in cells, animals, and some human studies [25,84].

Clinical data and latest reviews therefore emphasize precursor strategies, not dietary GSH, as the most effective approach for raising body GSH [84,86,91,102]. Diets rich in precursors and phytochemicals (e.g., Mediterranean style, brassica vegetables, polyphenol-rich foods, omega-3-rich fish) are associated with higher plasma GSH or improved GSH-related redox balance [25,86,91]. In this way, overall dietary patterns, such as those rich in fruits, vegetables, and whole grains, can support GSH levels due to their high content of antioxidants and precursors [25,84]. These interventions can help manage oxidative stress and support overall health. Therefore, most scientists are of the opinion that diets rich in lean protein, brassica vegetables, polyphenol-rich fruits/vegetables, green tea, and omega-3-rich foods are a low-cost, safe way to support GSH status [25,86]. However, current evidence that dietary GSH itself meaningfully increases systemic GSH in humans is limited and described as a possibility that requires more clarification [25,85].

#### 3.2.3. Glutathione Supplements and Precursors: How They Raise Body Glutathione

The same trend can be seen in the case of dietary supplements. As studies have proven, targeted oral GSH formulation can acutely increase blood GSH, but this is a short-term effect [85,103,104]. Some recent reviews note that most ingested GSH is degraded by intestinal γ-glutamyltransferase, making direct oral GSH not the most efficient option, whereas supplying its amino acid precursors or other synthesis activators clearly enhances tissue GSH synthesis [86]. All GSH supplementation strategies and their effects are presented in Table 3.

Human data consistently show low oral bioavailability of standard GSH. Reviews and clinical analyses note that conventional oral GSH does not reliably raise plasma or erythrocyte GSH and is generally considered therapeutically weak when given via the gastrointestinal route [103,105,111]. A meta-analysis of clinical trials found no significant increase in erythrocyte or plasma GSH with standard oral doses overall, though 500–1000 mg/day for 6 months or longer may increase body stores in some contexts [104], while some longer clinical trials show dose-dependent increases in body GSH and reduced oxidative stress at 250–1000 mg/day over 6 months [25].

It has been proven that supplementation with a combination of GSH precursors (glutamine, α-ketoglutarate, alanine, glycine) for 8 weeks significantly increased GSH levels and improved redox status in healthy adults [109]; additionally, oral γ-glutamylcysteine, the immediate GSH precursor, increased lymphocyte GSH levels in humans [85]. GlyNAC (glycine + NAC) corrected intracellular GSH deficiency and multiple aging-related abnormalities in older adults in a pilot trial [109]. Multiple reviews, animal studies and clinical trials conclude that supplementing precursors (*N*-acetylcysteine, glycine, other amino acids) is generally more effective than oral GSH for boosting endogenous synthesis [106,107,108,109,110,111,112,113,114,115].

Some studies have shown that oral GSH (up to 500–1000 mg per day) is generally well tolerated [104,116]. Typical side effects are mild and may include symptoms such as gas and bloating [116]. In animal studies, GSH did not show genotoxicity even at high doses (1500 mg/kg/day) [117]. GSH can be taken by both healthy individuals and those with certain medical conditions. In older adults and people with type 2 diabetes, GSH or GlyNAC supplements improved redox markers and certain metabolic parameters without significant safety concerns [42,91,107,118,119].

Since GSH can protect healthy tissues but may also protect tumor cells and alter the effects of chemotherapy, cancer patients are not recommended to take this supplement on their own without the supervision of an oncologist [120]. Data from long-term studies on GSH use during pregnancy and in children are limited; therefore, it is recommended to use this supplement with caution and consult a doctor [25,85,118].

### 3.3. Absorption, Metabolism, and Bioavailability of Oral GSH

Although there are many sources and supplements of GSH on the market, oral administration of GSH remains one of the biggest challenges in harnessing its therapeutic potential. The oral bioavailability of GSH is exceptionally low, which substantially limits its clinical efficacy when administered through conventional oral routes [121]. Even when foods contain high levels of GSH, this does not necessarily translate to increased GSH levels in the body. Reviews and clinical analyses note that conventional oral GSH does not reliably raise plasma or erythrocyte GSH and is generally considered therapeutically weak when given via the gastrointestinal route [92,121,122]. This discrepancy between GSH content and its systemic availability is a critical distinction that has often been overlooked in nutritional discussions. GSH is a tripeptide, and like all peptides and proteins, its oral bioavailability is inherently limited. The standard oral bioavailability of native GSH has been reported to be below 1% [123].

#### 3.3.1. Barriers to Oral GSH Absorption

The poor oral bioavailability of GSH stems from multiple interconnected physiological and biochemical barriers within the gastrointestinal tract [92,121,122,123]. The primary challenge involves enzymatic degradation: GSH is rapidly broken down by various peptidases and proteases present throughout the gastrointestinal system, particularly in the intestinal lumen, and by enterocyte enzymes. Additionally, as a tripeptide, GSH exhibits poor permeability across the intestinal epithelial membrane due to its hydrophilic nature and size [122]. The physicochemical properties of GSH create unfavorable conditions for passive absorption, the predominant pathway for most nutrients and bioactive compounds in the gastrointestinal tract. These factors collectively result in minimal systemic exposure following oral administration, rendering standard oral formulations largely ineffective for achieving therapeutic blood levels [92].

#### 3.3.2. Metabolism of GSH in the Liver

Once absorbed, GSH undergoes phase II metabolism, with conjugation reactions representing a major metabolic pathway [6,7,8]. For the small amount of intact GSH that does manage to reach systemic circulation, the liver becomes the next critical site of metabolism. The liver is the primary organ for GSH homeostasis and interorgan GSH transfer. However, the liver also contains high levels of GSH-metabolizing enzymes with particularly high hepatic glutathionase expression. The degradation products (glutamate, cysteine, and glycine) are then available for hepatic reutilization or systemic distribution [7,8].

Normally, hepatocytes synthesize GSH *de novo* and release a portion of it into the blood to maintain plasma GSH levels. However, this represents newly synthesized GSH, not dietary GSH [10,92]. Understanding these metabolic pathways is crucial for developing formulations that optimize both absorption and systemic retention of active GSH or its therapeutic metabolites.

#### 3.3.3. Alternative Delivery Approaches and Enhancement Strategies

While gastrointestinal absorption of GSH is poor, research has demonstrated that absorption from the oral mucosa—particularly the orobuccal (sublingual and buccal) mucosa—is substantially superior to traditional oral delivery [122,124]. When absorbed through the orobuccal mucosa, GSH passes directly into systemic circulation via the rich network of blood vessels in this region, bypassing hepatic first-pass metabolism and achieving much higher absorption rates compared to gastrointestinal uptake [123,124]. This route has garnered clinical interest, particularly for applications requiring rapid systemic exposure or when high bioavailability is essential for therapeutic efficacy.

Recognizing the limitations of conventional oral GSH delivery, researchers and pharmaceutical developers have explored numerous strategies to improve its bioavailability [123]. These approaches include co-administration with penetration enhancers and enzymatic inhibitors that can temporarily reduce proteolytic degradation in the gastrointestinal tract. Encapsulation technologies represent another major strategy, with GSH being loaded into protective carriers such as nanoparticles, microemulsions, and liposomes that shield the peptide from enzymatic degradation [125,126,127]. Chemical modification represents another innovative approach: recent studies have demonstrated that *N*-methylation of GSH—specifically the *N*-methylation of the cysteine residue—can substantially enhance resistance to enzymatic degradation while maintaining antioxidant activity [121,123]. Studies of a methylated GSH analogue revealed a remarkable 16.8-fold increase in plasma half-life and a 16.1-fold increase in oral bioavailability compared to native GSH [123].

#### 3.3.4. Practical Recommendations for Maintaining Adequate GSH

The scientific literature identifies three approaches for supporting GSH: dietary intake, endogenous synthesis stimulation, and supplementation [128]. Evidence indicates that consuming GSH-containing foods (fresh vegetables, legumes, mushrooms, fresh meats, and seafood) provides direct dietary GSH [69,70,71,72,73,74,75,76,77]. However, current evidence that dietary GSH itself meaningfully increases systemic GSH in humans is limited. Diets rich in GSH precursor amino acids (cysteine, glycine, glutamate) and phytochemicals (from brassica vegetables, polyphenol-rich fruits, green tea, omega-3-rich fish) activate GSH synthesis pathways and demonstrate more consistent increases in body GSH than dietary GSH alone [25,84,87].

Precursor-based supplements (NAC, glycine, GlyNAC combinations) and Nrf2-activating compounds show stronger and more consistent GSH elevation than direct oral GSH supplementation. Precursor supplementation appears safe at standard doses for most adults but may not be appropriate for cancer patients without oncologist supervision. For the general population, maintaining GSH is best achieved through a diet rich in sulfur-containing amino acids and Nrf2-activating compounds (e.g., sulforaphane). In clinical settings where endogenous synthesis is compromised (e.g., advanced age or chronic metabolic stress), precursor-based strategies like GlyNAC currently offer more consistent evidence for improving intracellular stores than standard oral GSH. Further long-term human trials are required to validate the safety and efficacy of high-dose supplementation.

## 4. Conclusions

Glutathione (GSH) is a vital antioxidant that supports numerous bodily functions, including detoxification, immune regulation, and mitochondrial health. Its role in reducing oxidative stress makes it beneficial for preventing and managing chronic diseases and promoting overall health. GSH is a vital neuroprotective agent in the brain and its ability to mitigate oxidative stress and support neuronal health makes it a promising target for therapeutic strategies against neurodegenerative diseases. GSH can be effective as a skin-lightening agent due to its anti-melanogenic and antioxidant properties. Research supports GSH to improve several hallmarks and risk factors of aging in older adults; however, definitive human evidence for disease prevention or lifespan extension remains absent.

For most individuals, prioritizing a diet rich in lean protein, sulfur-containing foods, brassica vegetables, polyphenol-rich produce, and omega-3-rich fish, combined with targeted amino acid precursors when indicated, provides a practical, evidence-based, and cost-effective approach to supporting GSH levels. Long-term prospective human clinical trials are needed to clarify the effects of GSH-enhancing interventions on chronic disease prevention and aging outcomes.

## Figures and Tables

**Table 1:** Food Sources of Glutathione.

Food Source	GSH Content (mg Per 100 g)	Reference(s)
*Plant foods*		
Mushrooms, dried	11.00–241.00	[69]
Legumes (mung beans, black beans, kidney beans, pinto beans, etc.)	15.00–37.00	[70,71]
Spinach	9.62–28.90	[72,73]
Asparagus	10.73–21.80	[73]
Avocado	10.42–20.60	[72]
Squash, zucchini	8.40–11.40	
Potatoes (baked or boiled)	10.20–11.00	[72]
Broccoli, brussels sprouts	1.90–10.00	
Carrots, tomatoes	5.90–7.50	[73]
Strawberries, grapefruit, cantaloupe,	6.10–6.90	
Green, red sweet peppers, garlic	3.40–5.50	
Nectarines, peaches, melons, watermelons	4.90–5.00	
Oranges, lemons, papaya, mangoes	4.18–4.80	
Cauliflower, cucumbers	3.78–4.00	
Bananas, pears, nuts and seeds, walnuts	3.30–3.70	
*Meat, subproducts*		
Chicken breast	36.26	[74]
Veal cutlet	26.30	[73]
Pork	13.70–18.90	
Beef, liver (chicken)	11.80–15.34	[73] [75]
Chicken	6.50–7.70	[73]
*Fish, seafood*		
*O. Niloticus*	245.86	[76]
*C. Crangon*	169.03	[77]
*B. amazonicus,* heart	83.59	[78]
*C. harengus membras*	73.76	[77]
*M. saxatilis,* heart	49.17	[79]
*P. maxima*	39.34	[77]
*Merluccius merluccius*	23.00	[80]
*B. amazonicus,* muscle	17.83	[77]
*Morone saxatilis*, striped bass (skeletal muscle)	12.29	[79]
*Austropotamobius torrentium, Astacus astacus*,		[81]
*Orconectes limosus*	6.76–8.30	[73]
Fish (cod and perch), pan-fried	5.70	[82]

**Table 2:** Foods Boosting Endogenous Synthesis of GSH.

Food	Mechanism of Action	Reference(s)
Foods rich in specific proteins that contain amino acids cysteine, glycine, glutamate	These amino acids are direct precursors for GSH synthesis, enhancing tissue GSH levels.	[12,25,86]
Garlic, onions	Contains sulfur compounds such as S-allylcysteine that enhance GSH levels. Onion extracts and flavonoids like quercetin can increase intracellular GSH levels by activating the gamma-glutamylcysteine synthetase promoter, which is essential for GSH synthesis.	[87]
Brassica vegetables (sulforaphane), lipoic acid	Nrf2 activators in the body; Nrf2 upregulates enzymes for GSH synthesis and cystine uptake; Nrf2 activators increase GSH in many cell types	[25]
Omega-3 fatty acids	Modulate the GSH network by activating Nrf2 and boosting synthesis and GPx activity in many normal tissues	[88]
GSH and resveratrol precursors	Increase endogenous levels of vitamins C, E, and A, enhancing antioxidant activity.	[89]
Plant species *Allium hookeri*	Rich in organosulfur compounds, it increases GSH levels and regulates glucose metabolism.	[45]
Spirulina, turmeric	These foods increase antioxidant enzymes and GSH levels, reducing oxidative stress.	[90]
Green tea (especially EGCG-rich polyphenols)	increases GSH by activating Nrf2 signaling, up-regulating GSH-related enzymes, and enhancing antioxidant and detoxification pathways	[91,92]
Milk	Milk containing A2 β-casein can promote the production of GSH in humans.	[93]
Nuts and seeds (particularly baru almonds and Brazil nuts)	Source of selenium, a key component of GSH peroxidase, can increase GSH levels in the body by enhancing the activity of GSH peroxidase.	[94,95]
Legumes, e.g., lentils, chickpeas (*Cicer arietinum L*)	Provide protein and may aid in the synthesis of GSH. Contain compounds that enhance the activity of antioxidant enzymes, including GSH reductase and GSH peroxidase. They express glutaredoxin, a protein that works with GSH to reduce oxidative stress and glutaredoxin helps maintain GSH levels. Chickpeas contain bioactive compounds like selenium and isoflavonoids, which can enhance the activity of GSH peroxidase.	[96,97,98,99]
Legumes like *Medicago falcata* and *Medicago truncatula*	Reach of nitric oxide (NO), which plays a crucial role in regulating GSH synthesis and influences the expression of genes involved in GSH synthesis, such as γ-glutamylcysteine synthetase and GSH synthetase.	[100]

**Table 3:** Different Supplementation Strategies and GSH Response.

Strategy/Context	Effect on Systemic or Intracellular GSH	Reference(s)
Conventional oral GSH (capsules)	Poorly bioavailable; often minimal systemic effect; inferior to sublingual GSH and NAC in one trial	[103,104]
Sublingual GSH	Clearly increased plasma total and reduced GSH, improved GSH/GSSG vs oral GSH and NAC	[104]
NAC (single or short-term oral dosing)	Raises cysteine; supports GSH synthesis when demand is high (paracetamol load, HIV, oxidative stress) but may not increase GSH in unstressed plasma	[105,106,107]
GlyNAC (glycine + NAC) in older adults	Corrects RBC GSH deficiency, improves redox and mitochondrial function in deficient states; in healthy elders, no overall increase in circulating GSH, but responders with high oxidative stress showed higher GSH	[108,109,110]
Oral precursor mix (cystine, glutamate, glycine), 3 months supplementation	GSH precursors’ supplement increased human GSH levels across test groups	[111]
Mixed GSH precursor supplements (cystine/cysteine, glutamate, glycine ± polydatin)	Increased erythrocyte/plasma GSH and other reduced thiols in healthy humans after weeks of supplementation	[104,112]
NAC or glutamine in HIV+	Both increased plasma GSH, normalizing it toward controls, via different precursor pools	[113]

## Data Availability

Not applicable.

## Abbreviations

The following abbreviations are used in this manuscript:

GlyNAC	Glycine and *N*-Acetylcysteine
GSH	Glutathione
NAC	*N*-acetylcysteine
Nrf2	Nuclear factor erythroid-2-related factor 2
RNS	Reactive nitrogen species
ROS	Reactive oxygen species
